# Identification of a New Virulent Clade in Enterohemorrhagic *Escherichia coli* O26:H11/H- Sequence Type 29

**DOI:** 10.1038/srep43136

**Published:** 2017-02-23

**Authors:** Nozomi Ishijima, Ken-ichi Lee, Tomomi Kuwahara, Haruyuki Nakayama-Imaohji, Saori Yoneda, Atsushi Iguchi, Yoshitoshi Ogura, Tetsuya Hayashi, Makoto Ohnishi, Sunao Iyoda

**Affiliations:** 1 Department of Bacteriology I, National Institute of Infectious Diseases (NIID), Tokyo 162-8640, Japan; 2 Department of Microbiology, Faculty of Medicine, Kagawa University, Kagawa 761-0793, Japan; 3 Department of Animal and Grassland Sciences, Faculty of Agriculture, University of Miyazaki, Miyazaki 889-2192, Japan; 4 Department of Bacteriology, Faculty of Medical Sciences, Kyushu University, Fukuoka 812-8582, Japan

## Abstract

Enterohemorrhagic *Escherichia coli* (EHEC) O26 infections cause severe human diseases such as hemolytic uremic syndrome and encephalopathy, and is the predominant serogroup among non-O157 EHEC in many countries. Shiga toxin (Stx), which consists of two distinct types (Stx1 and Stx2), plays a central role in EHEC pathogenesis. The major *stx* gene type in EHEC O26 strains is *stx1*, although isolates with only *stx2* have emerged in Japan since 2012 and have been reported in Europe. In this study, we selected 27 EHEC O26 strains isolated in Japan and identified a distinct genetic clade within sequence type (ST) 29, designated ST29C1, that carried only *stx2* and had the plasmid gene profile *ehxA*+/*katP*−/*espP*+/*etpD*−. We showed that ST29C1 strains produced higher Stx2a levels, and greater virulence in Vero cells and in germ-free mice than other lineages. We also showed that ST29C1 was a distinct phylogenetic clade by SNP analysis using whole genome sequences and clearly differed from the major European EHEC O26 virulent clone, which was designated ST29C2 in this study. The combination of toxin production analysis, virulence analysis in Vero cells and germ-free mice, and phylogenetic analysis identified a newly emerging virulent EHEC clade.

Enterohemorrhagic *Escherichia coli* (EHEC) is an important category of diarrheagenic *E. coli*. EHEC strains are responsible for causing diarrhea and also severe diseases, such as hemolytic uremic syndrome (HUS) and encephalopathy^1^,^2^. Shiga toxin (Stx) is the most critical EHEC virulence factor and is required for the severe symptoms. There are two major Stx groups, Stx1 and Stx2, which have been further divided into several subtypes: subtypes *stx1a, stx1c* and *stx1d* for the *stx1* gene, and subtypes *stx2a* to *stx2g* for the *stx2* gene^3^.

Over 3,000 cases of EHEC infections, including asymptomatic carriers, have been reported annually in Japan over the last two decades. Among the EHEC isolates in Japan during 2009–2014, serogroup O157 (serotype O157:H7/H- [non motile]) strains were predominant, and among non-O157 EHEC strains, serogroup O26 (serotype O26:H11/H-) strains were predominant. Most (92%) EHEC O26:H11/H- strains carried only the *stx1* gene, while 1.31% carried only the *stx2* gene and 5.78% carried both *stx1* and *stx2* genes^4^,^5^,^6^,^7^,^8^,^9^,^10^,^11^. However, the prevalence of EHEC O26:H11/H- isolates carrying only *stx2* increased in 2012–2014 in Japan^4^,^5^,^6^,^7^,^8^,^9^,^10^,^11^ and, as has been reported in Europe, a new EHEC O26:H11/H- clone carrying only *stx2* has emerged and has been associated with severe cases^12^,^13^,^14^. Multi–locus sequence typing (MLST) analysis showed that the European EHEC O26:H11 strains that have been subtyped were either sequence type (ST) 21 or ST29, with ST21 consisting of strains carrying either only *stx1a*, only *stx2a*, or both *stx1a* and *stx2a*, and ST29 consisting of strains carrying only *stx2a*^12^,^13^,^14^. The large EHEC plasmid encoding virulence-related factors, such as enterohemolysin (*ehxA*), catalase peroxidase (*katP*), serine protease (*espP*), and an effector of the type II protein secretion system (*etpD*), has been found in most EHEC O26:H11/H- strains. The pattern of presence or absence of these virulence-related genes indicated that many European EHEC O26 ST21 strains (52 of 67 strains) exhibited an *ehxA*+/*katP*+/*espP*+/*etpD*− plasmid gene profile, and most ST29 strains (65 of 69 strains) had a distinctive *ehxA*+/*katP*−/*espP*−/*etpD*+ plasmid gene profile^12^.

For the present study, we selected 27 EHEC O26 strains carrying *stx2a* that were isolated from epidemiologically unlinked cases during 2007–2014 in Japan and analyzed these strains by MLST and large plasmid gene profiling. The strains were further analyzed for their Stx2a expression level, Vero cell cytotoxicity, and mouse virulence. We also carried out whole genome sequence (WGS)-based phylogenetic analysis of the 27 EHEC O26 strains and compared those results with data for other EHEC O26 strains found in a public database. From these analyses, we identified a new genetic clade among EHEC O26:H11/H- ST29 strains, designated ST29C1, which does not include the European EHEC O26 virulent clone.

## Results

### MLST analysis of *stx2*-positive EHEC O26:H11/H- strains

EHEC strains carrying only *stx1* have been the major type of EHEC O26:H11/H- in Japan^4^,^5^,^6^,^7^,^8^,^9^,^10^,^11^. However, the number of EHEC O26 strains carrying only *stx2* has been increasing since 2012 as shown in Table 1. We performed multiple comparison by Fisher’s exact test with Holm’s adjusted *p* values, and found that the proportion of severe disease cases was significantly higher in the strains carrying only *stx2* or both *stx1* and *stx2* than the strains carrying only *stx1* (adjusted *p* value < 0.05), as has been reported previously in Europe^12^,^13^,^14^. Therefore, we carried out genotypic and phenotypic characterization of *stx2*-positive EHEC O26:H11/H- strains isolated in Japan during 2007–2014. From *stx* subtyping results, we selected 16 strains carrying only *stx2a* and 11 strains carrying both *stx1a* and *stx2a* for studies to compare the Stx2a level among strains. These 27 strains are listed in Supplementary Table S1 and were further characterized as described below.

We next determined the sequence types of these strains by MLST. All 11 strains carrying both *stx1a* and *stx2a* and 7 strains carrying only *stx2a* were classified as ST21, while the 9 other strains carrying only *stx2a* were classified as ST29 (Table 2). One of the strains carrying only *stx2a* had a different *mdh* allele (allele number 375) from that of other ST29 strains (Supplementary Table S2) and was classified as a new ST, designated ST5172. Since ST5172 was a one- and two-locus variant of ST29 and ST21, respectively, it was considered to be a new member of the ST29 complex (Supplementary Table S2).

### Comparison of Stx2 expression levels in different ST and *stx* types

**Figure 1:**
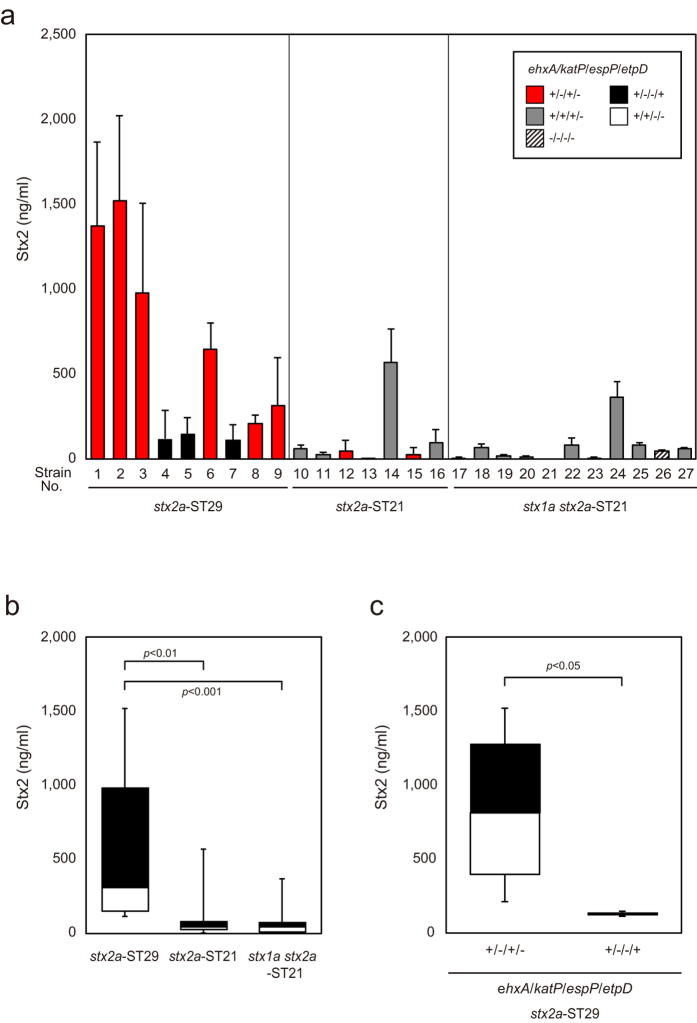
Quantitation of released Stx2 levels. Stx2 levels released into culture supernatants by strains 1 to 27 (Supplementary Table S1) were measured by sandwich-ELISA. Each value was the result of at least three independent experiments. (**a**) Stx2 values are shown as the mean ± SD (error bars). (**b**) The amount of released Stx2 was plotted according to the *stx* type and ST. The boxes show the 25th to 75th percentiles, with values from the 25th percentile to the median white and values from the median to the 75th percentile black. The bars show the minimum and maximum values. (**c**) Amount of released Stx2 of *stx2*-ST29 strains with different plasmid gene profiles. The data are plotted as in (**b**).

To elucidate the phenotypic differences between ST21 and ST29 strains carrying different *stx* types, Stx2a expression levels were determined by sandwich-ELISA. As shown in Fig. 1a, the Stx2a level in mitomycin C-induced culture supernatants of several, but not all, ST29 strains were higher than other strains. Statistical analysis showed that the Stx2 level of ST29 strains was significantly higher than those of ST21 strains (Fig. 1b). To further characterize the genotype of these strains, we carried out PCR analysis to detect four genes (*ehxA, katP, espP*, and *etpD*) commonly carried on a large virulence plasmid in most EHEC O26:H11/H- strains. All strains were found to have plasmid gene profiles *ehxA*+/*katP*−/*espP*+/*etpD*−, *ehxA*+/*katP*−/*espP*−/*etpD*+, *ehxA*+/*katP*+/*espP*+/*etpD*−, *ehxA*+/*katP*+/*espP*−/*etpD*−, or *ehxA*−/*katP*−/*espP*−/*etpD*− (Table 2). The plasmid gene profiles of ST29 strains were either *ehxA*+/*katP*−/*espP*+/*etpD*− or *ehxA*+/*katP*−/*espP*−/*etpD*+ (Table 2): the former profile was not found in any European O26 ST29 strain and the latter was the major profile in European O26 ST29 strains^12^,^13^,^14^,^15^. We noted that all the ST29 strains with the *ehxA*+/*katP*−/*espP*+/*etpD*− gene profile had significantly higher Stx2 expression levels than ST29 strains with the *ehxA*+/*katP*−/*espP*−/*etpD*+ gene profile (Fig. 1c). In contrast to these results, the ST21 strains with the *ehxA*+/*katP*−/*espP*+/*etpD*− gene profile (strain 12 and 15) did not show higher Stx2a expression levels than the ST21 strains with other plasmid gene profiles (Fig. 1a). These results together suggest that Stx2a high producing strains may belong to a specific genetic lineage within ST29 with the *ehxA*+/*katP*−/*espP*+/*etpD*− plasmid profile, which was further pursued later in this study. We also carried out reversed passive latex agglutination (RPLA) (VTEC-RPLA: Denka Seiken Co. Ltd., Tokyo, Japan) to determine the Stx2 levels in bacterial culture supernatants of strains grown in medium with and without mitomycin C. RPLA titer of strains without mitomycin C showed similar expression levels as those with mitomycin C (Supplementary Fig. S1). Therefore, all further studies were done in cultures with mitomycin C.

We next measured *stx2a* mRNA levels using the same culture conditions as those used for analyzing Stx2a levels, to investigate whether the different Stx2a levels were due to different levels of *stx2a* gene transcription. Several *stx2a*-ST29 strains had higher *stx2a* mRNA levels than other strains (Supplementary Fig. S2a). The mean *stx2a* mRNA level was higher in *stx2a*-ST29 strains than in *stx2a*-ST21 and *stx1a stx2a*-ST21 strains (Supplementary Fig. S2b). Therefore, the high level of Stx2a expression in *stx2a*-ST29 strains was due to a high level of *stx2a* transcription in these strains. We then compared *stx2* mRNA levels between *stx2a*-ST29 strains with the *ehxA*+/*katP*−/*espP*+/*etpD*− and *ehxA*+/*katP*−/*espP*−/*etpD*+ gene profiles. The mean *stx2a* mRNA level of *ehxA*+/*katP*−/*espP*+/*etpD*− strains was higher than that of *ehxA*+/*katP*−/*espP*−/*etpD*+ strains, although the difference was not statistically significant (Supplementary Fig. S2c). This discrepancy may have been due, in part, to the results that several strains (number 1–3) produced high levels of Stx2a but only moderate levels of *stx2a* mRNA.

### Stx2 expression and Vero cell cytotoxicity

**Figure 2:**
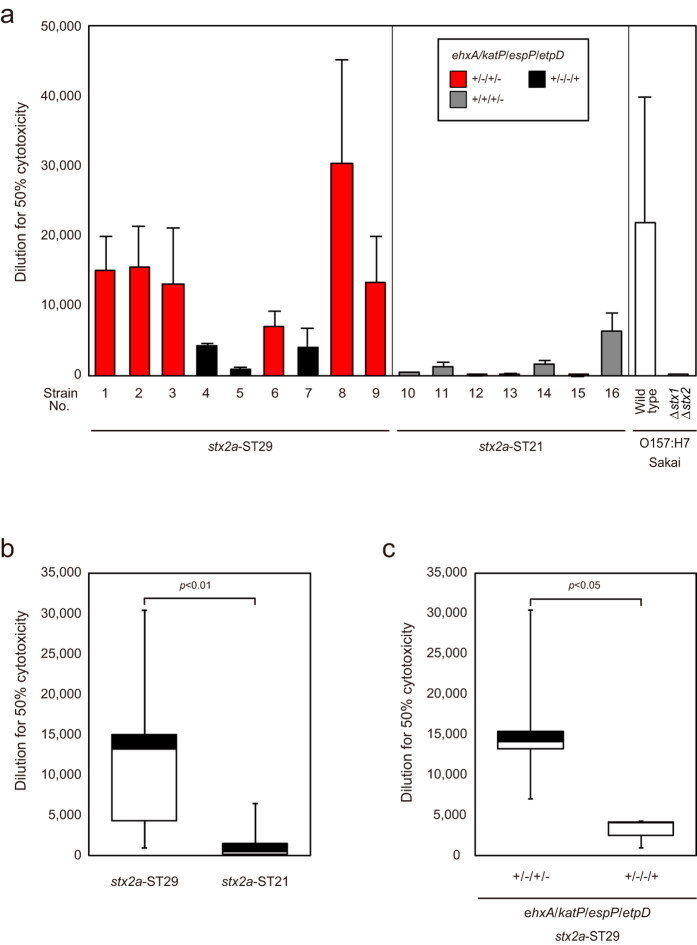
Vero cell cytotoxicity of EHEC O26 strains. The culture supernatant dilution to produce 50% cytotoxicity was calculated. Strains 1 to 27 are listed in Supplementary Table S1. The figures are data from three independent experiments. (**a**) The data are shown as the mean ± SD (error bars). (**b**) The culture supernatant dilution to produce 50% cytotoxicity was plotted according to the *stx* and ST types. The boxes show the 25th to 75th percentiles, with values from the 25th percentile to the median white and values from the median to the 75th percentile black. The bars show the minimum and maximum values. (**c**) The culture supernatant dilution to produce 50% cytotoxicity of *stx2*-ST29 strains was plotted according to the plasmid gene profile.

To investigate whether different Stx2a expression levels contributed to EHEC virulence, we carried out Vero cell cytotoxicity assays. The culture supernatant dilution to produce 50% cytotoxicity was compared between ST21 and ST29 strains, and between ST29 strains with the *ehxA*+/*katP*−/*espP*+/*etpD*− and *ehxA*+/*katP*−/*espP*−/*etpD*+ gene profiles. The cytotoxicity of ST29 strains was higher than for ST21 strains (Fig. 2a and b), and was higher for ST29 strains with the *ehxA*+/*katP*−/*espP*+/*etpD*− gene profile than for ST29 strains with the *ehxA*+/*katP*−/*espP*−/*etpD*+ gene profile (Fig. 2c), which was consistent with the data for Stx2 expression levels (Fig. 1).

### Phylogenetic analysis of the EHEC O26:H11/H- strains in this study using whole genome sequence data

**Figure 3:**
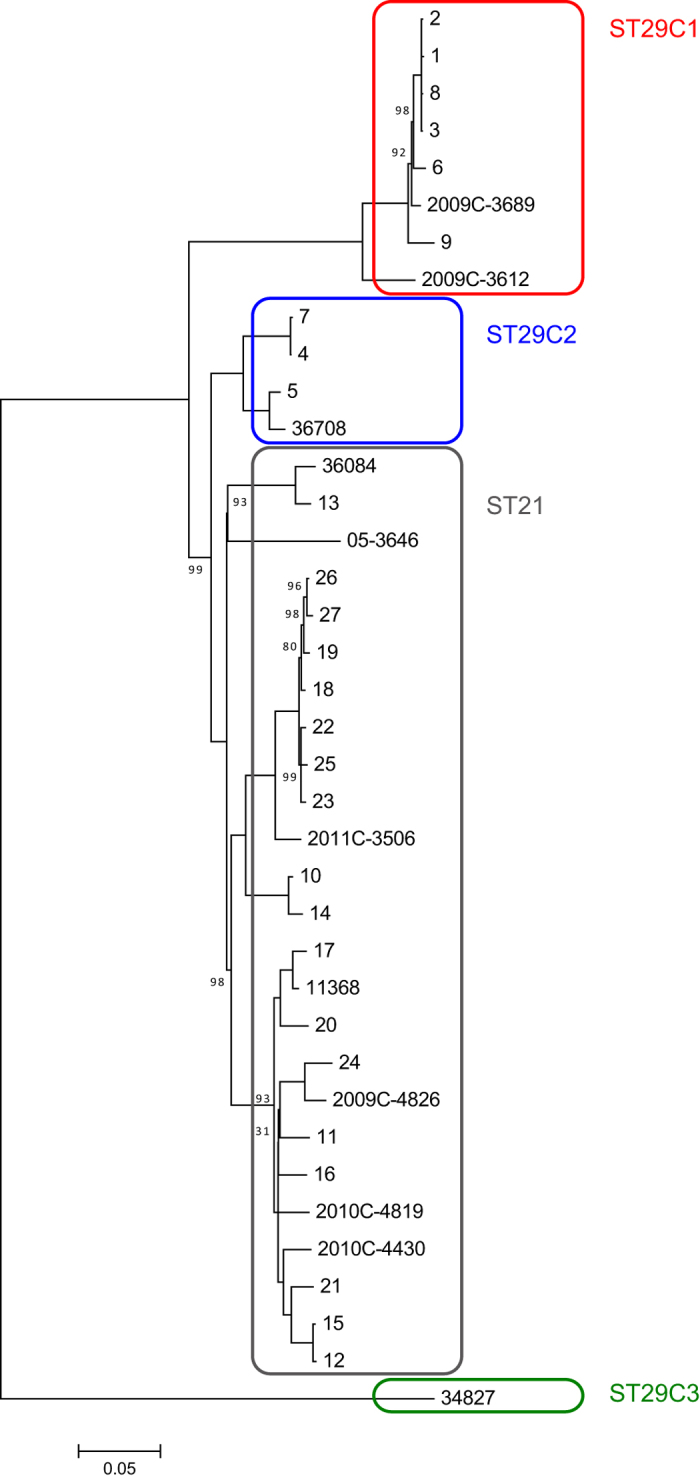
Phylogenetic analysis of EHEC O26 strains in this study. A maximum likelihood phylogenetic tree was reconstructed with MEGA 7, based on 3,593 SNP sites in the genome backbone, which were identified using a genome sequence comparison among 37 EHEC O26 strains. Strains 1 to 27 are listed in Supplementary Table S1. The reliability of the tree’s internal branches was assessed by bootstrapping with 1,000 pseudoreplicates. The scale bar shows the number of substitutions per site. A bootstrap value of 100 was omitted in this figure. Strain 11368 was used as a reference. EHEC O26 strains in ST21, ST29C1, ST29C2, and ST29C3 are in gray, red, blue, and green boxes, respectively.

Phylogenetic analysis using WGSs of the strains in this study and the genome sequences of some database strains (strains listed in Supplementary Table S1) was carried out to determine the relationships among these strains. The phylogenetic tree indicated that ST29 strains could be divided into three clades, designated ST29C1, ST29C2 and ST29C3 (Fig. 3). The clade pairwise SNP distances were much higher between clades than within the same clade, which supported the clade designations (Supplementary Table S4). Clade ST29C1 consisted of six strains isolated in Japan and two strains (2009C-3689 and 2009C3612) isolated in the USA^16^, all with the *ehxA*+/*katP*−/*espP*+/*etpD*− plasmid gene profile. Clade ST29C2 was closely related to ST21 and consisted of four strains with the *ehxA*+/*katP*−/*espP*−/*etpD*+ plasmid gene profile: three of these were Japanese strains 4,5, and 7 (Fig. 3). In addition to these three Japanese strains, there was one ST29 strain, 36708, that was isolated in France and was similar to the new European virulent new clone. Clade ST29C3 consisted of a single EHEC O26 strain, 34827, that was isolated in France. In addition, four other ST29 strains carrying *stx2a* or *stx2d*^17^ were grouped together as ST29C3 (data not shown). In these strains, the four plasmid genes were not detected in our *in silico* analysis as described previously^14^. In contrast to these results, the ST21 strains in this study were genetically homogeneous compared to the ST29 strains.

### Virulence assays of O26 strains in mice

**Figure 4:**
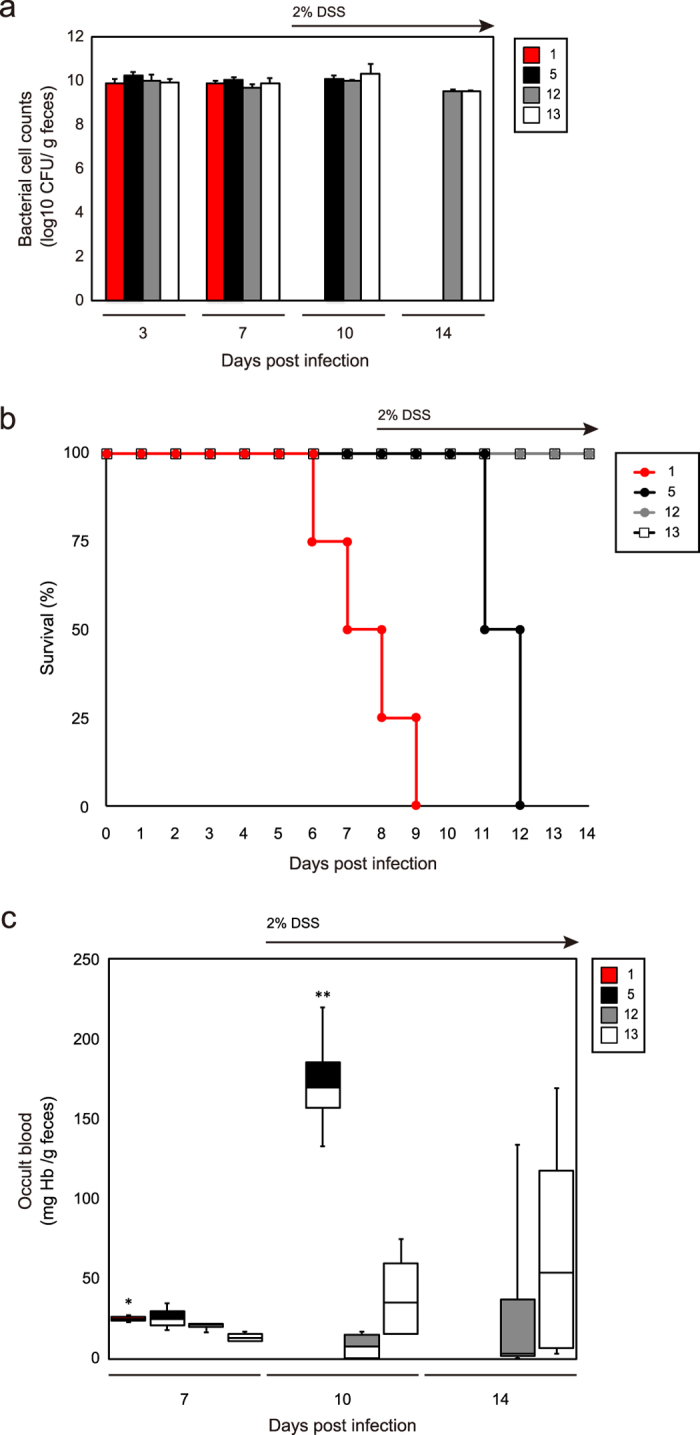
Virulence assays of EHEC O26 strains in germ-free mice. (**a**) Bacterial cell counts of EHEC O26 strains 1, 5, 12, and 13 in mouse feces. (**b**) Kaplan-Meier survival curves of infected mice. Log-rank test results: 1 vs. 5, *p* = 0.0202; 5 vs. 12 or 13, *p* = 0.0180. (**c**) Fecal occult blood of infected mice. *p < 0.05, vs. 13; **p < 0.01, vs. 12 or 13.

To investigate the virulence of the clades in this study in mice, four O26:H11/H- strains were selected as follows: one ST29C1 strain (strain 1) with the *ehxA*+/*katP*−/*espP*+/*etpD*− gene profile, one ST29C2 strain (strain 5) with the *ehxA*+/*katP*−/*espP*−/*etpD*+ gene profile, and two ST21 strains (strains 12 and 13) carrying *stx2a* and with the *ehxA*+/*katP*−/*espP*+/*etpD*− or the *ehxA*+/*katP*+/*espP*+/*etpD*− gene profile, respectively. Each strain was inoculated into four germ-free BALB/cA mice orally and virulence and mouse mortality was examined. As shown in Fig. 4a, bacterial cell counts in mouse feces did not differ significantly among surviving mice infected with the four EHEC O26 strains.

ST29C1 strain 1 showed the highest virulence of the strains studied (Fig. 4b), with two of four mice dying before addition of 2% (w/v) dextran sodium sulfate (DSS) to induce colitis in the mouse bowel. Furthermore, ST29C1 strain 1 showed statistically significantly greater virulence than ST29C2 strain 5 (Fig. 4b). Quantitative analysis of fecal occult bloods showed that ST29C2 strain 5 showed higher levels of fecal occult blood than ST21 strains 12 and 13 (Fig. 4c). These results together indicate that ST29C1 strains 1 had the greatest virulence in mice of the strains studied, and ST29C2 was less virulent than ST29C1 strain 1, but more virulent than ST21 strains 12 and 13.

## Discussion

A previous study showed that EHEC O26:H11/H- strains carrying only *stx2a* have a significant potential to cause HUS, and that strains carrying both *stx1a* and *stx2a* have a similar potential to cause both HUS and diarrhea without HUS^12^. In addition, EHEC O26 ST21 and ST29 strains carrying *stx2a* do not differ substantially in their association with HUS^12^. From these results, it was concluded that the presence of *stx2a* rather than the ST21/29 subtype was a predictor for HUS development in EHEC O26-infected individuals^12^. In the present study, we characterized the EHEC O26 strains carrying only *stx2a* and identified a new phylogenetic clade, designated ST29C1, among the ST29 strains. ST29C1 strains had higher levels of Stx2a *in vitro* than ST29C2 and ST21 strains. We further showed that, the virulence phenotypes of all ST29C1 strains against Vero cells and at least one strain of ST29C1 against germ-free mice were higher than those of other tested EHEC O26 strains. The various virulence phenotypes including toxin expression levels of infected bacterial pathogens are one of the requirements for clinical manifestations in infected persons^1^,^2^. In this study, we conclude that the members of the phylogenetic clade ST29C1 are associated with high virulence with EHEC O26. However, to see whether the phylogenetic clade ST29C1 is also one of the requirements for causing severe diseases in patients, it is desirable to proceed with further epidemiological investigations.

ST29C1 strains have a unique plasmid gene profile, *ehxA*+/*katP*−/*espP*+/*etpD*−, and the ST29C1 clade does not include any EHEC O26 European strains, including the new highly virulent clone^14^. However, from our WGS-based phylogenetic analysis comparing the strains in this study with EHEC O26 strains isolated in countries other than Japan, we identified two strains (2009C-3689 and 2009C-3612; Fig. 3) isolated in the USA^18^ that were also in clade ST29C1. Therefore, ST29C1 is not a specific clone of EHEC O26:H11/H- in Japan. We also showed that three ST29C2 strains were similar to the new European EHEC O26 clone. In the germ-free mouse infection model in this study, one ST29C2 strain was shown to be significantly more virulent than two ST21 strains, even though the increased levels of Stx2a expression and Vero cell cytotoxicity were not significantly different between ST29C2 and the ST21 strains. Therefore, ST29 strains may have additional virulence potential including virulence genes other than *stx2a*. We have now isolated ST29-specific sequences not found in ST21 strains to test this possibility in a future study.

An SNP-based subtyping method has classified EHEC O157:H7/H- strains into 9 genetic clades (clades 1–9). Of these clades, clade 8 strains were shown to be significantly associated with HUS cases^19^,^20^. Although several clade 8 strains overexpressed Stx2 compared to clade 1–3 strains, this was not found for all clade 8 strains^21^. A recent study indicated that clade 8 strains could be subdivided into two distinct subclades, clade 8a and clade 8b^22^. Of these subclades, clade 8a carried a specific Stx2a-converting bacteriophage, which may be responsible for the higher expression level of Stx2 in this strain^22^. Therefore, the ST29C1 strain in this study may carry similar Stx2 phage sequences. A further study to analyze Stx2a-converting phage sequences, including the insertion site in the bacterial genome, may elucidate the cause of higher Stx2a expression in ST29C1 strains compared to other lineages.

In this study, we demonstrated that the combination of phenotypic analysis examining for toxin production, virulence in Vero cells and germ-free mice, and phylogenetic analysis using WGS identified a newly emerging virulent EHEC clade in EHEC O26:H11/H- ST29.

## Material and Methods

### Bacterial strains

The EHEC O26:H11/H- and O157:H7 strains used in this study are summarized in Supplementary Table S1. We selected 27 EHEC O26:H11/H- strains that were epidemiologically unrelated, with 16 strains carrying only the *stx2* gene and 11 carrying both the *stx1* and *stx2* genes. SKI-5142 is a Lac-negative derivative of EHEC O157:H7 strain Sakai, as described previously^23^, and was used as a wild-type EHEC O157:H7 strain in this study. SKI-5500 is an *stx1-stx2* double deletion mutant derivative of SKI-5142, as described previously^24^. These two strains were used as controls in Vero cell cytotoxicity assays.

### PCR

The *stx1* and *stx2* subtypes were identified by PCR carried out as described previously^3^. Virulence-related genes *ehxA, katP, espP* and *etpD* carried on an EHEC large plasmid were detected by PCR as described previously^25^,^26^,^27^,^28^.

### MLST analysis

MLST was carried out as described previously^29^. Briefly, seven housekeeping genes (*adk, fumC, gyrB, icd, mdh, purA* and *recA*) were sequenced and alleles and STs were assigned in accordance with the *E. coli* MLST database at the University of Warwick ( http://mlst.ucc.ie/mlst/mlst/).

### Bacterial sample preparation

Bacterial samples were prepared for analysis of Stx2 expression levels and cell cytotoxicity as follows. EHEC strains were grown in Luria-Bertani (LB) medium at 37 °C without shaking. Overnight cultures were diluted 1:50 in fresh LB medium. After shaking for 2 h at 37 °C, the optical density at 600 nm was adjusted to 0.6 by dilution with LB medium. After further growth for 3 h at 37 °C in LB medium with 0.5 μg mitomycin C (Sigma-Aldrich, MO, USA)/ml, the culture was centrifuged at 10,000 × g for 5 min to pellet the cells. The supernatants were filtered through 0.45 μm filters (Merck Millipore, Darmstadt, Germany), and the filtrates were analyzed by sandwich-ELISA, cytotoxicity assays and RPLA. The cell pellets were used to purify total RNA as described below.

### Real-time PCR

Total RNA was purified from bacterial cell pellets using ISOGEN II (Nippon gene, Tokyo, Japan) and treated with TURBO DNase (Thermo Fisher Scientific, MA, USA). cDNA was synthesized from 1 μg samples of total RNA using random primers and reverse transcriptase ReverTra Ace (TOYOBO, Osaka, Japan). Real-time PCR for measuring *stx2* mRNA expression levels was carried out using a KAPA SYBR Fast LightCycler 480 Readymix Kit (KAPA Biosystems, MA, USA) with Light Cycler nano (Roche Diagnostic, Basel, Switzerland). mRNA expression was calculated relative to *rpoA* gene expression, as an endogenous reference standard. Primers RT-stx2 F (5′-CGACCCCTCTTGAACATA-3′) and RT-stx2 R (5′-TAGACATCAAGCCCTCGTAT-3′)^30^ were used to amplify the *stx2* gene, and rpoA_1F (5′-GAACGCCTTCTTTGGTGCTG-3′) and rpoA_1R (5′-GCTTTGGCCATACTCTGGGT-3′) were used to amplify the *rpoA* gene.

### Sandwich-ELISA

Sandwich-ELISA was used to detect Stx2 in the bacterial culture supernatants, and was carried out in RIDASCREEN Verotoxin microtiter plates (R-Biopharm GmbH, Darmstadt, Germany) coated with capture antibodies for detecting both Stx1 and Stx2. A monoclonal antibody against Stx2 (LSBio, WA, USA) conjugated with horseradish peroxidase using Peroxidase Labeling Kit–NH_2_ (Dojindo, Kumamoto, Japan) was used as the detection antibody. A standard curve was determined with 2-fold serial dilutions of purified Stx2 (provided by Denka Seiken Co. Ltd.). Detection was carried out with urea peroxidase/TMB substrate reagent and 1 M sulfuric acid as the stop reagent, both were provided in the RIDASCREEN Verotoxin kit. Absorbance at 450 nm (A_450_) was measured using a 96-well plate reader (Multiscan FC, Thermo Fisher Scientific, MA, USA).

### Vero cell cytotoxicity assays

Vero cells were maintained in Dulbecco’s modified Eagle’s minimum essential medium (DMEM) (Thermo Fisher Scientific, MA, USA) supplemented with 10% (v/v) heat-inactivated fetal bovine serum (GE Healthcare UK Ltd., Buckinghamshire, UK). The cytotoxicity of bacterial culture supernatants against Vero cells was determined by the method of Gentry and Dalrymple^31^ with slight modification. Ten microliter samples of 10-fold serial dilutions of cell-free culture supernatants and 90 μl of a trypsinized Vero cell suspension in DMEM with 10% FCS were added to 96-well plates (1 × 10^4^ cells/well). After 72 h incubation at 37 °C in the presence of 5% CO_2_, the relative number of live cells was determined with a Cell Counting Kit-8 (Dojindo). In this assay, a water-soluble tetrazolium salt, WST-8, was reduced by the intracellular dehydrogenase activity to produce an orange formazan dye. The amount of the formazan dye (A_450_) was proportional to the number of live cells. After addition of 10 μl of the Cell Counting Kit-8 solution, the 96-well plates were incubated for 1 h at 37 °C in the presence of 5% CO_2_ and A_450_ was measured with a 96-well plate reader. EHEC O157:H7 Sakai strain (SKI-5142) and its mutant derivative with both *stx1* and *stx2* genes deleted (SKI-5500) were used as control strains.

### Phylogenetic analysis of EHEC O26:H11/H- strains

To analyze the phylogenetic relationships among the strains in this study, WGSs were determined using a MiSeq (Illumina, San Diego, CA, USA). Genomic DNA libraries were prepared using Nextera XT DNA sample prep kits (Illumina). The pooled libraries were analyzed by multiplex paired-end sequencing (300 × 2 bp). The sequence reads were assembled using the A5-miseq pipeline^32^. In addition to the strains isolated in Japan, WGSs of 10 EHEC O26 strains isolated in the USA and France (Supplementary Table S3) were included in the phylogenetic analyses. Contig sequences of 37 EHEC O26 strains were aligned with the EHEC O26 11368 genome sequence (GenBank accession number: AP010953) using MUMmer version 3.2259^33^ to identify the conserved backbone of these strains and SNP sites. A 3,915,864 bp sequence of the EHEC O26 11368 genome was conserved in all the strains examined, with >99% sequence identity and a >2,000 bp alignment length. Recombinogenic regions were deleted by RecHMM^34^. Phylogenetic relationships were then determined by reconstruction of a phylogenetic tree using the maximum likelihood method, based on the Tamura-Nei model with 1,000 bootstraps^35^, using MEGA software^36^ and the concatenated alignment of 3,593 SNP sites located in the conserved backbone. The short reads and contigs used in this study have been deposited under BioProject PRJDB5136.

### Mouse virulence assays

We selected a minimum strain set for a mouse virulence assay based on data obtained in *in vitro* experiments above, for reducing the number of mouse used in a virulence assays from the point of view of animal welfare. EHEC O26 strains (strains 1, 5, 12 and 13) were incubated in Brain Heart Infusion (BHI; Eiken Chemical Co. Ltd., Tokyo, Japan) broth with shaking at 37 °C for 16 h. The bacterial culture was diluted in sterile saline, and orally administered to four germ-free BALB/cA mice (6–9 wk old, two males and two females; CLEA Japan Inc., Tokyo, Japan), with 2.06–3.24 × 10^7^ colony-forming units/mouse. To simulate hemorrhagic colitis, a filter-sterilized 2% DSS solution was given as drinking water after the 7th day of the inoculation. Viable bacteria in the feces were counted by culturing saline-dissolved feces (collected at 3, 7, 10, and 14 days after inoculation) for 24 h on BHI agar plates. The mice were euthanized 7 days after the 2% DSS treatment or at the time mice became moribund.

### Fecal occult blood measurements

Fecal occult blood of EHEC O26-infected mice was quantified by heme-catalyzed oxidation of tetramethylbenzidine by hydrogen peroxide^37^. In brief, 50 μl samples of fecal suspensions (0.1 mg/ml) in distilled water were boiled for 10 min and then mixed with 0.3 ml of 30% acetic acid and 0.45 ml of ethyl acetate. After centrifugation, the supernatants were collected and 0.1 ml of 0.6 mM tetramethylbenzidine solution was added. Finally, 50 μl of 3% hydrogen peroxide or distilled water (as a blank) was added to the samples, and absorbance at 660 nm and 450 nm was measured at 30 sec and 60 sec after addition of hydrogen peroxide. Human hemoglobin (Sigma) was used as a standard. Fecal occult blood concentrations were calculated as described by Welch *et al*.^37^.

### Statistical Analysis

Statistical analyses were performed using Mann-Whitney *U* test (GraphPad Prism ver. 6.03 for Windows: GraphPad Software Inc., CA, USA) for quantification of Stx2/*stx2* levels and Vero cell cytotoxicity assays, and ANOVA and Tukey’s post-hoc test (StatFlex ver. 6.0: Artech Co., Ltd., Tokyo, Japan) for bacterial cell counts in mouse feces and fecal occult blood. Kaplan–Meier survival curves were generated and log-rank tests were performed. Data were considered to be significantly different if the *p* value was less than 0.05.

### Ethics statement

Animal experiments were carried out in accordance with Japanese legislation (Act on Welfare and Management of Animals, 1973, revised in 2012) and guidelines under the jurisdiction of the Ministry of Education, Culture, Sports, Science and Technology, Japan (Fundamental Guidelines for Proper Conduct of Animal Experiment and Related Activities in Academic Research Institutions, 2006). The protocols of animal experiments were approved by the Animal Care and Use Committee for Kagawa University (Approval Number; 15102). Animal care, housing, feeding, sampling, observation, and environmental enrichment were performed in accordance with the guidelines.

## Additional Information

**How to cite this article:** Ishijima, N. *et al*. Identification of a New Virulent Clade in Enterohemorrhagic *Escherichia coli* O26:H11/H- Sequence Type 29. *Sci. Rep.* **7**, 43136; doi: 10.1038/srep43136 (2017).

**Publisher's note:** Springer Nature remains neutral with regard to jurisdictional claims in published maps and institutional affiliations.

## Supplementary Material

Supplementary Information

## Figures and Tables

**Table 1:** Number of EHEC O26:H11/H- isolates in Japan during 2009–2014.

Year	Year	Number of isolates with *stx* type (% of severe disease cases†)		
	*stx1*	*stx2*	*stx1 stx2*	
2009	645 (16.3)	1 (100.0)	55 (23.6)
2010	520 (18.7)	1 (100.0)	44 (38.6)
2011	658 (13.5)	1 (100.0)	129 (26.4)
2012	767 (11.2)	20 (25.0)	30 (43.3)
2013	844 (15.9)	46 (19.6)	31 (29.0)
2014	760 (17.9)	15 (40.0)	15 (40.0)
Total	4,194 (15.4)	84 (27.4)	304 (30.3)

^†^Defined as patients developing bloody diarrhea, kidney failure, HUS, and encephalopathy. Adjusted *p* values: *stx1* vs *stx2*, 0.011*; *stx1* vs *stx1 stx2*, 0.0000000017*; *stx2* vs *stx1 stx2*, 0.686. *Considered to be significantly different if the *p* value < 0.05.

**Table 2:** Plasmid gene profiles of O26:H11/H- strains carrying *stx2a*.

*stx* genotype	Sequence type	Strain no.	*ehxA*	*katP*	*espP*	*etpD*
*stx2a*	ST29	1, 2, 3, 6, 8	+	−	+	−
*stx2a*	ST29	4, 5, 7	+	−	−	+
*stx2a*	ST5172	9	+	−	+	−
*stx2a*	ST21	10, 11, 13, 14, 16	+	+	+	−
*stx2a*	ST21	12, 15	+	−	+	−
*stx1a stx2a*	ST21	17, 18, 19, 20, 22, 24, 25, 27	+	+	+	−
*stx1a stx2a*	ST21	21	+	+	−	−
*stx1a stx2a*	ST21	23, 26	−	−	−	−
