# Supplementary material for: Identification of a New Virulent Clade in Enterohemorrhagic Escherichia coli O26:H11/H- Sequence Type 29
Source: Sci Rep. 2017 Feb 23;7:43136. doi: 10.1038/srep43136 (PMC5322567; doi:10.1038/srep43136)
Supplement: Supplementary Information [file srep43136-s1.pdf]

Supplementary Information

Identification of a New Virulent Clade in Enterohemorrhagic *Escherichia coli* O26:H11/H- Sequence Type 29

Nozomi Ishijima[1], Ken-ichi Lee[1], Tomomi Kuwahara[2], Haruyuki Nakayama-Imaohji[2], Saori Yoneda[2], Atsushi Iguchi[3], Yoshitoshi Ogura[4], Tetsuya Hayashi[4], Makoto Ohnishi[1] and Sunao Iyoda[1]*

[1] Department of Bacteriology I, National Institute of Infectious Diseases (NIID), Tokyo 162-8640, Japan

[2] Department of Microbiology, Faculty of Medicine, Kagawa University, Kagawa 761-0793, Japan

[3] Department of Animal and Grassland Sciences, Faculty of Agriculture, University of Miyazaki, Miyazaki 889-2192, Japan

[4] Department of Bacteriology, Faculty of Medical Sciences, Kyushu University, Fukuoka 812-8582, Japan

* Corresponding author

E-mail: siyoda@nih.go.jp

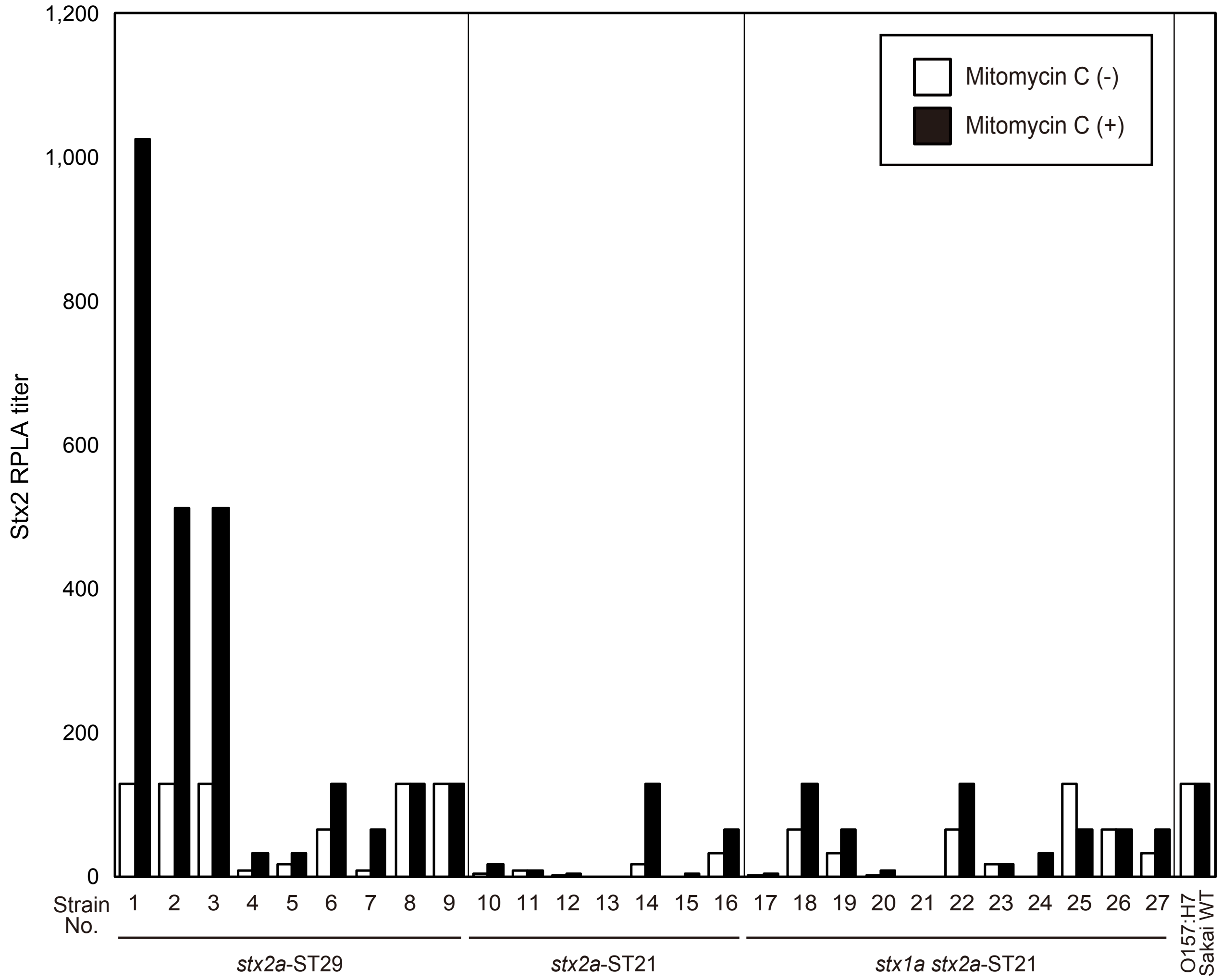

Supplementary Figure S1. Comparison of the expression levels of Stx2 in EHEC O26 strains grown with or without mitomycin C. RPLA for Stx2 was carried out in O26 strains according to manufacturer's instructions. The strains are listed in Supplementary Table S1.

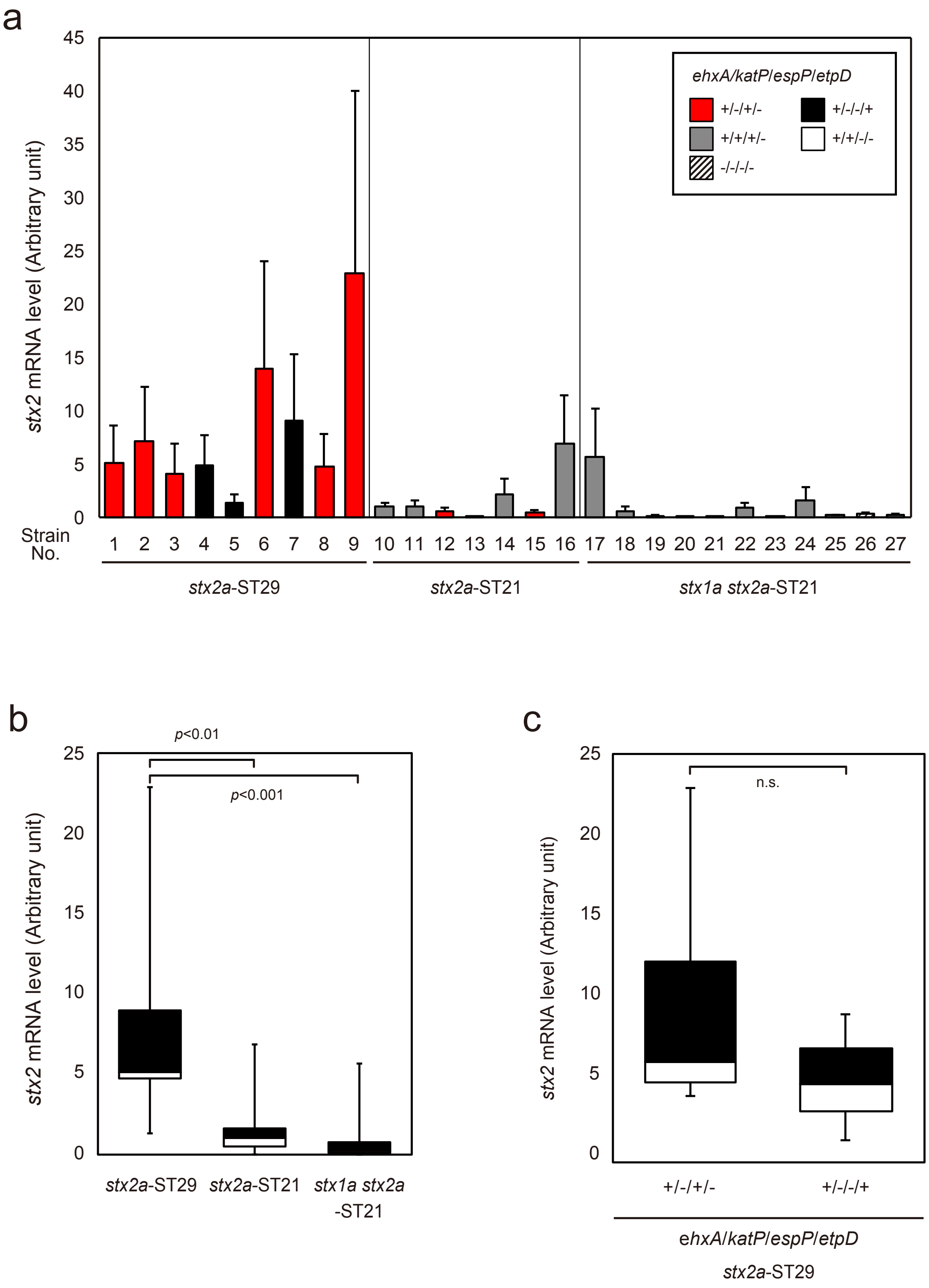

Supplementary Figure S2. *stx2* mRNA expression levels. The amount of *stx2* mRNA in each EHEC O26 strain cell pellet was analyzed by real-time PCR. Strains 1 to 27 are listed in Supplementary Table S1. Each result was from at least three independent experiments. (a) Each value is the mean ± SD (error bars). (b) The *stx2* mRNA data are plotted as in Fig. 1b. (c) The *stx2* mRNA data of *stx2*-ST29 strains with different plasmid gene profiles. The data are plotted as in (b). n.s., not significant.

Supplementary Table S1. EHEC strains used in this study.

Strain no.	Serotype [H genotype]	Serial strain no. in this study	Year of isolation or reference no.	Disease association*
2602-1	O26:H11	1	2013	BD
2602-2	O26:H11	2	2013	BD
2602-3	O26:H11	3	2013	BD
2602-4	O26:H- [H11]	4	2012	HUS
2602-5	O26:H11	5	2008	BD
2602-6	O26:H11	6	2010	D
2602-7	O26:H- [H11]	7	2013	BD
2602-8	O26:H11	8	2013	unknown
2602-9	O26:H11	9	2014	unknown
2602-10	O26:H11	10	2007	D
2602-11	O26:H11	11	2007	BD
2602-12	O26:H11	12	2012	D
2602-13	O26:H11	13	2013	D
2602-14	O26:H11	14	2012	BD
2602-15	O26:H11	15	2013	unknown
2602-16	O26:H11	16	2014	BD
2612-17	O26:H11	17	2012	D
2612-18	O26:H- [H11]	18	2010	BD
2612-19	O26:H11	19	2012	D
2612-20	O26:H11	20	2013	D
2612-21	O26:H11	21	2008	none
2612-22	O26:H11	22	2008	BD
2612-23	O26:H11	23	2009	D
2612-24	O26:H11	24	2011	BD
2612-25	O26:H- [H11]	25	2011	BD
2612-26	O26:H11	26	2011	D
2612-27	O26:H11	27	2013	BD
SKI-5142	O157:H7 Sakai Δ(*lacIZYA*)	Wild Type	Ref. 23	
SKI-5500	SKI-5142 Δ*stx1*Δ*stx2*	Δ*stx1*Δ*stx2*	Ref. 24	

* HUS, Hemolytic uremic syndrome; B, Bloody diarrhea; D, Diarrhea

Supplementary Table S2. Multi–locus sequence typing of O26:H11/H- strains.

Sequence type	Housekeeping gene allele						
	adk	*fumC*	*gyrB*	*icd*	*mdh*	*purA*	*recA*
ST29	6	4	12	16	9	7	7
ST5172	6	4	12	16	375	7	7
ST21	16	4	12	16	9	7	7

Supplementary Table S3. EHEC strains used for phylogenetic analysis based on whole genome sequences*.

Strain	Country	GenBank accession no.	*stx* subtype	ST	Presence of plasmid genes			
					ehxA	*katP*	*espP*	*etpD*
34827	France	LDXB00000000	*stx2a*	29	-	-	-	-
36084	France	LDXI00000000	*stx2a*	21	+	+	+	-
36708	France	LDXG00000000	*stx2a*	29	+	-	-	+
05-3646	USA	JHOE00000000	*stx1a*	21	+	+	-	-
2009C-3612	USA	JHGZ00000000	*stx2a*	29	+	-	+	-
2009C-3689	USA	JHGX00000000	*stx2a*	29	+	-	+	-
2009C-4826	USA	JHGI00000000	*stx1a*	21	+	+	+	-
2010C-4430	USA	JHND00000000	*stx1a*	21	+	+	+	-
2010C-4819	USA	JHMP00000000	*stx1a stx2a*	21	+	+	+	-
2011C-3506	USA	JHLS00000000	*stx1a*	21	+	+	+	-

**stx* subtypes, MLST and the presence of plasmid genes were determined by *in silico* analyses.

Supplementary Table S4. Mean values of pairwise SNP distances within and between groups of strains.

Group	Mean SNP distance (range)			
	Within group	Between groups		
		ST21	ST29C1	ST29C2
ST21	261 (10-405)			
ST29C1	95 (5-261)	712 (629-782)		
ST29C2	278 (5-435)	372 (56-455)	695 (639-795)	
ST29C3	NA	1,239 (1,175-1,289)	1,367 (1,346-1,389)	1,220 (1,185-1,300)

NA, not applicable.
